# Fire as a driver and mediator of predator–prey interactions

**DOI:** 10.1111/brv.12853

**Published:** 2022-03-23

**Authors:** Tim S. Doherty, William L. Geary, Chris J. Jolly, Kristina J. Macdonald, Vivianna Miritis, Darcy J. Watchorn, Michael J. Cherry, L. Mike Conner, Tania Marisol González, Sarah M. Legge, Euan G. Ritchie, Clare Stawski, Chris R. Dickman

**Affiliations:** ^1^ School of Life and Environmental Sciences, Heydon‐Laurence Building A08 The University of Sydney Sydney NSW 2006 Australia; ^2^ Biodiversity Strategy and Knowledge Branch, Biodiversity Division Department of Environment, Land, Water and Planning 8 Nicholson Street East Melbourne VIC 3002 Australia; ^3^ Centre for Integrative Ecology, School of Life and Environmental Sciences (Burwood Campus) Deakin University 75 Pigdons Road Waurn Ponds VIC 3216 Australia; ^4^ School of Agricultural, Environmental and Veterinary Sciences Charles Sturt University Gungalman Drive Albury NSW 2640 Australia; ^5^ School of Natural Sciences, G17 Macquarie University 205B Culloden Road Macquarie Park NSW 2109 Australia; ^6^ Caesar Kleberg Wildlife Research Institute Texas A&M University‐Kingsville 700 University Boulevard, MSC 218 Kingsville TX 78363 U.S.A.; ^7^ The Jones Center at Ichauway 3988 Jones Center Drive Newton GA 39870 U.S.A.; ^8^ Laboratorio de Ecología del Paisaje y Modelación de Ecosistemas ECOLMOD, Departamento de Biología, Facultad de Ciencias Universidad Nacional de Colombia Edificio 421 Bogotá 111321 Colombia; ^9^ Fenner School of Environment & Society The Australian National University Linnaeus Way Canberra ACT 2601 Australia; ^10^ Centre for Biodiversity Conservation Science University of Queensland Level 5 Goddard Building St Lucia QLD 4072 Australia; ^11^ Department of Biology Norwegian University of Science and Technology Trondheim NO‐7491 Norway; ^12^ School of Science, Technology and Engineering University of the Sunshine Coast Maroochydore DC QLD 4558 Australia

**Keywords:** carnivore, foraging behaviour, hunting behaviour, interaction, landscape of fear, mega‐fire, multiple threats, predation rates, prescribed burning, wildfire

## Abstract

Both fire and predators have strong influences on the population dynamics and behaviour of animals, and the effects of predators may either be strengthened or weakened by fire. However, knowledge of how fire drives or mediates predator–prey interactions is fragmented and has not been synthesised. Here, we review and synthesise knowledge of how fire influences predator and prey behaviour and interactions. We develop a conceptual model based on predator–prey theory and empirical examples to address four key questions: (*i*) how and why do predators respond to fire; (*ii*) how and why does prey vulnerability change post‐fire; (*iii*) what mechanisms do prey use to reduce predation risk post‐fire; and (*iv*) what are the outcomes of predator–fire interactions for prey populations? We then discuss these findings in the context of wildlife conservation and ecosystem management before outlining priorities for future research. Fire‐induced changes in vegetation structure, resource availability, and animal behaviour influence predator–prey encounter rates, the amount of time prey are vulnerable during an encounter, and the conditional probability of prey death given an encounter. How a predator responds to fire depends on fire characteristics (e.g. season, severity), their hunting behaviour (ambush or pursuit predator), movement behaviour, territoriality, and intra‐guild dynamics. Prey species that rely on habitat structure for avoiding predation often experience increased predation rates and lower survival in recently burnt areas. By contrast, some prey species benefit from the opening up of habitat after fire because it makes it easier to detect predators and to modify their behaviour appropriately. Reduced prey body condition after fire can increase predation risk either through impaired ability to escape predators, or increased need to forage in risky areas due to being energetically stressed. To reduce risk of predation in the post‐fire environment, prey may change their habitat use, increase sheltering behaviour, change their movement behaviour, or use camouflage through cryptic colouring and background matching. Field experiments and population viability modelling show instances where fire either amplifies or does not amplify the impacts of predators on prey populations, and *vice versa*. In some instances, intense and sustained post‐fire predation may lead to local extinctions of prey populations. Human disruption of fire regimes is impacting faunal communities, with consequences for predator and prey behaviour and population dynamics. Key areas for future research include: capturing data continuously before, during and after fires; teasing out the relative importance of changes in visibility and shelter availability in different contexts; documenting changes in acoustic and olfactory cues for both predators and prey; addressing taxonomic and geographic biases in the literature; and predicting and testing how changes in fire‐regime characteristics reshape predator–prey interactions. Understanding and managing the consequences for predator–prey communities will be critical for effective ecosystem management and species conservation in this era of global change.

## I INTRODUCTION

Fire affects ecosystems across the globe by consuming plant material, destroying and creating habitat, and altering resource availability, in turn shaping the abundance and distribution of plants, animals, fungi and other organisms (Bowman *et al*., 2009; He, Lamont & Pausas, 2019). Fires can result in extensive animal mortality (e.g. Erwin & Stasiak, 1979; Tomas *et al.,* 2021), but in many cases mortality rates are low and many animals survive the direct impacts of fire (Jolly *et al*., 2022). However, simply surviving the fire does not spell the end of the danger period; animals can be at heightened risk after fire due to reduced shelter and resource availability, and increased exposure to predators, with consequences for survival, reproduction and population dynamics (Sutherland & Dickman, 1999; Nieman *et al*., 2021).

Predators also play a key role in ecosystem function by influencing the behaviour and population dynamics of their prey and competitors (Creel & Christianson, 2008; Ripple *et al*., 2014), and fire may either strengthen or weaken these effects. Stronger effects of predators on prey can arise directly if predators are attracted to fires due to enhanced foraging opportunities (e.g. raptors hunting small prey fleeing the flames), whereas reduced effects may occur if predators are themselves killed in the flames. Fires also influence predator impacts indirectly by altering habitat structure, which is a key determinant of predation rates (Janssen *et al*., 2007) and prey foraging behaviour (Verdolin, 2006). Geary *et al*. (2020) found that predator responses to fire across the globe are diverse and differ among ecosystems, fire types, species and populations. Some predators respond positively to fire (e.g. higher occurrence in burnt areas; Birtsas, Sokos & Exadactylos, 2012), others show the opposite response (e.g. Santos & Poquet, 2010), and some show little response (e.g. Webb & Shine, 2008). Despite this heterogeneity, the most common driver used to explain predator responses to fire is food availability (Geary *et al*., 2020). There is also much evidence that fire influences the behaviour, abundance and distribution of common prey groups, including small (Griffiths & Brook, 2014) and large mammals (Fisher & Wilkinson, 2005; Ritchie *et al*., 2008), birds (Woinarski & Legge, 2013), amphibians (dos Anjos, Solé & Benchimol, 2021), reptiles (Hu, Doherty & Jessop, 2020), and invertebrates (Swengel, 2001; Chitwood *et al*., 2017). While fire clearly influences the behaviour and population dynamics of both predators and prey, the consequences for predator–prey *interactions* remain poorly understood and have not been synthesised. Knowledge of how fire affects hunting and foraging behaviour, predation rates, physiology, and population dynamics will help answer fundamental questions about how and why some species, but not others, persist in burnt landscapes (Pausas & Parr, 2018; Stawski & Doty, 2019; Nimmo *et al*., 2021).

Understanding how fire influences predator–prey interactions is relevant not only to fundamental ecology, but also to applied ecological problems, such as game and invasive species management, threatened species conservation, and fire management. Fire regimes across the globe have shifted from historical baselines due to displacement of Indigenous Peoples, land‐use and habitat change, prescribed burning and, increasingly, climate change (Russell‐Smith *et al*., 2003; Bowman *et al*., 2020; Pyne, 2020; Fletcher *et al*., 2021). In some places, fire has been reduced or completely excluded (e.g. Backer, Jensen & McPherson, 2004; Parsons & Gosper, 2011), while other locations are experiencing increases in fire size, frequency, or severity (e.g. Pausas & Fernández‐Muñoz, 2012). The global fire season lengthened by an average of 18.7% between 1979 and 2013 (Jolly *et al*., 2015), and catastrophic fires have recently impacted large areas in Brazil, Australia, the USA and many other locations (Kganyago & Shikwambana, 2020; Nolan *et al*., 2020). Potential consequences of altered fire regimes include vegetation state transitions (Dwomoh & Wimberly, 2017), increased extinction risk (Jones *et al*., 2016; Kelly *et al*., 2020), exotic species invasions (Reilly *et al*., 2020), changes in animal behaviour, physiology and health (Stawski *et al*., 2016; Álvarez‐Ruiz *et al*., 2021; Kay *et al*., 2021), and altered species interactions (Geary *et al*., 2018; Smith, 2018). Shifts in fire regimes that strengthen the effects of predators are particularly concerning because the combined impacts of these two processes could push some prey species towards local or complete extinction (Brooker & Brooker, 1994; Leahy *et al*., 2015; Whitehead *et al*., 2018).

Here, we synthesise knowledge of how fire influences predator–prey interactions, spanning vertebrates and invertebrates from a range of ecosystems globally. Drawing on predation risk theory, we develop a conceptual framework to answer the following questions: (*i*) how and why do predators respond to fire; (*ii*) how and why does prey vulnerability change post‐fire; (*iii*) what mechanisms do prey use to reduce predation risk post‐fire; and (*iv*) what are the outcomes of predator–fire interactions for prey populations? We discuss these findings in the context of ecosystem management and wildlife conservation, before detailing outstanding research questions and future directions for the field.

We sourced evidence through semi‐structured searches of *Scopus* and *Google Scholar* using combinations of the following key words: *fire*, burn*, predator*, predation, prey, predator–prey, fear and carnivor*. We also used a snowball sampling approach by inspecting the reference lists and citations of relevant studies. We further developed the evidence base by drawing on our expert knowledge spanning a range of taxonomic groups, countries, ecosystem types, and management regimes. Due to the broad research topic and the diverse nature of the available evidence (varying study designs and questions), we decided that a knowledge synthesis was more appropriate than a systematic review or meta‐analysis. Most of the available evidence relates to comparisons of burnt and unburnt habitat, although we discuss fire regime characteristics, such as fire frequency, season, and severity, where possible.

## II CONCEPTUAL FRAMEWORK

**Fig. 1:**
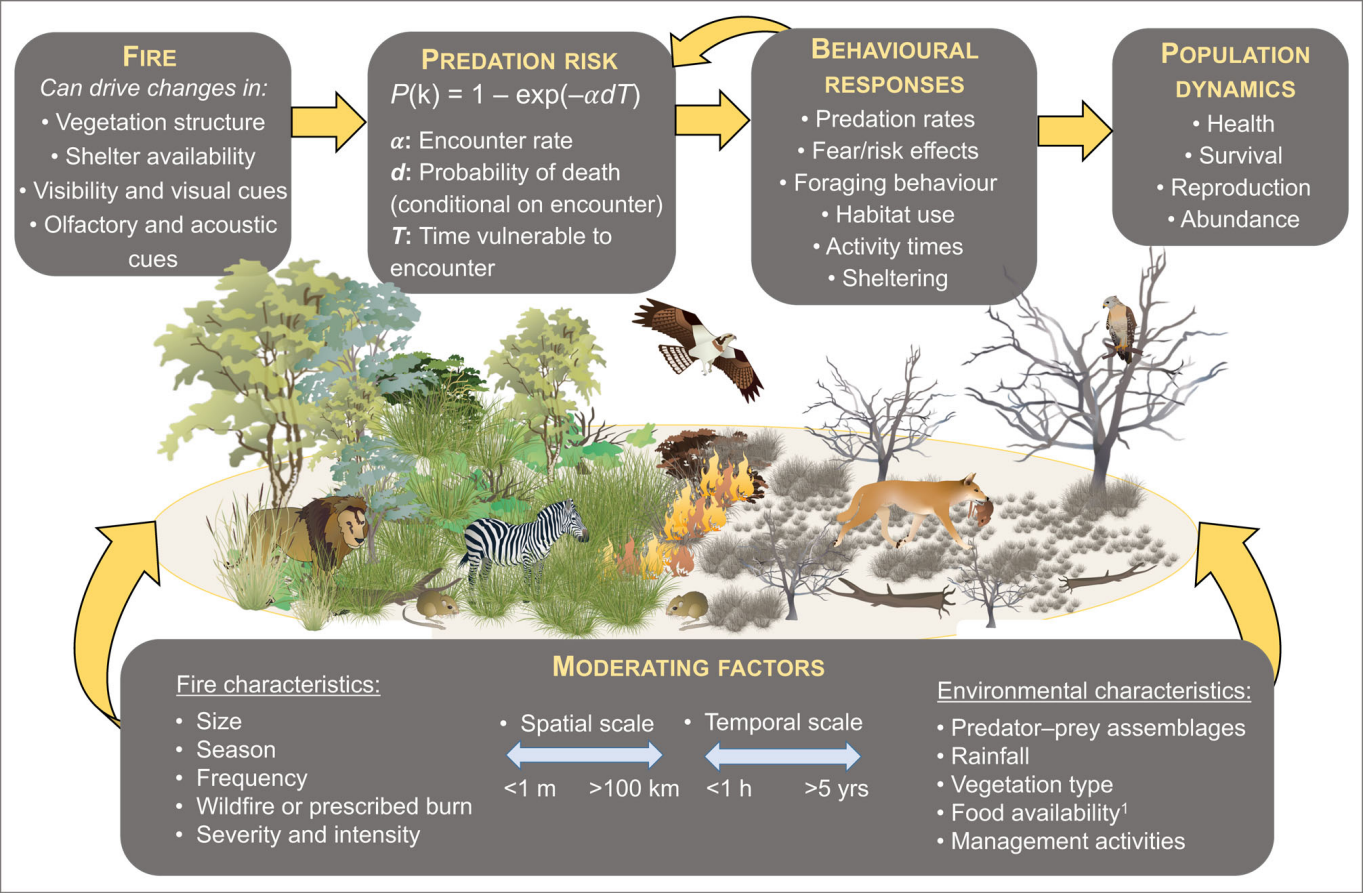
Conceptual framework illustrating how fire can drive changes in vegetation structure, shelter availability, visibility, and visual, olfactory and acoustic cues. These changes influence different components of predation risk (Lima & Dill, 1990), which in turn lead to behavioural responses by predators and prey, and consequences for population dynamics. Behavioural responses can feed back into the predation risk equation as predators and prey adjust to changes in one another's behaviour. The manifestation of these top‐level components can also be affected by moderating factors, including fire characteristics, rainfall, vegetation type, food availability, composition of predator and prey assemblages, management activities, and the spatial and temporal scales over which these factors operate. Images are courtesy of the Integration and Application Network (ian.umces.edu/media‐library) and the NESP Northern Australia Hub (nespnorthern.edu.au). ^1^Food availability for predators refers to alternative foods beyond the prey species in question.

**Fig. 2:**
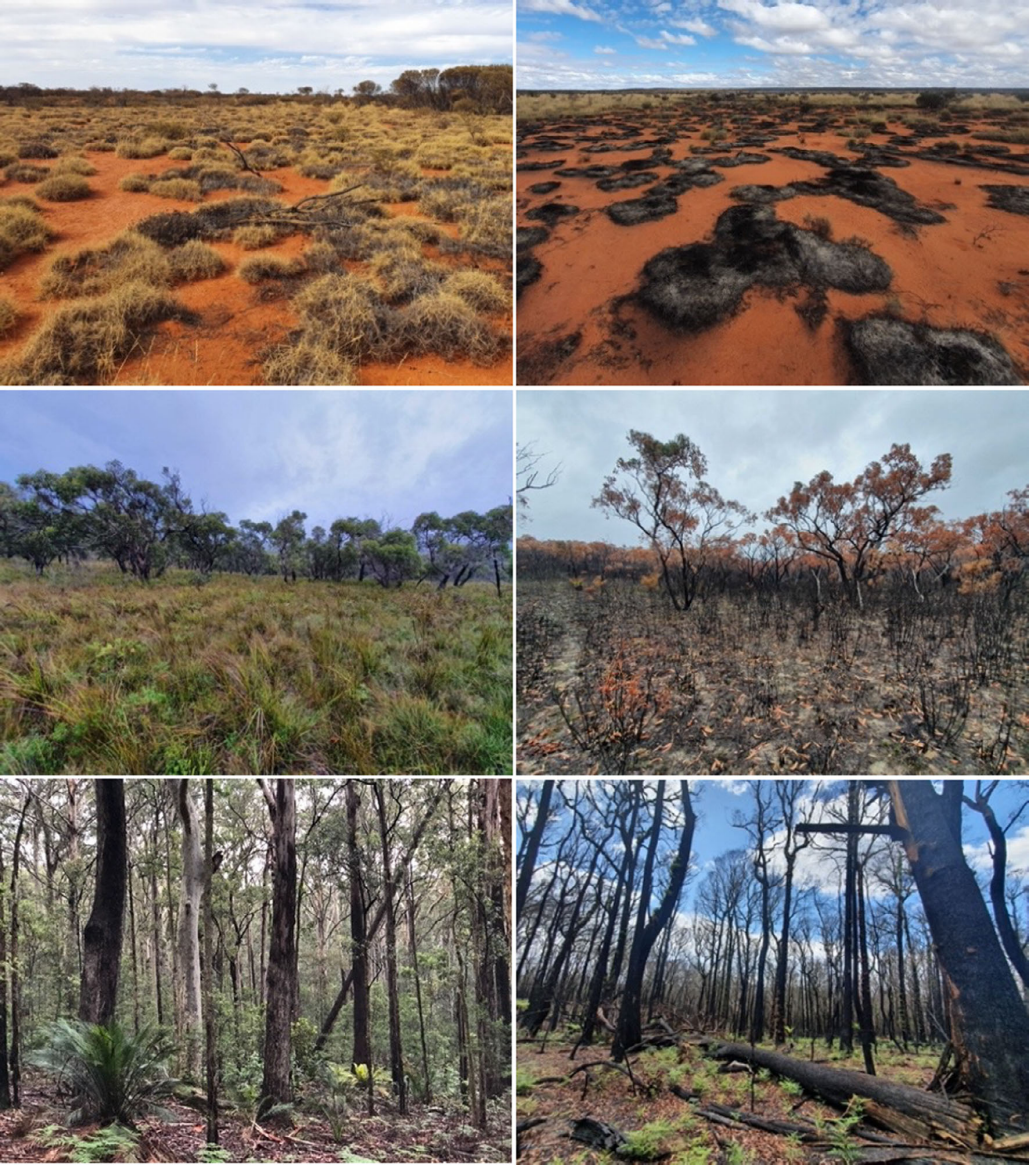
Unburnt and burnt grassland (top), woodland (middle), and forest (bottom). The grassland and woodland were burnt in planned burns, while the forest was burnt in a wildfire. Photograph credits: T. Doherty (top), D. Watchorn (middle) and V. Miritis (bottom).

The primary mechanism through which fire alters predator and prey behaviour is via changes in habitat structure (Fig. 1). Fire consumes live and dead plant material, generally causing a temporary simplification of habitat structure, with vegetation becoming sparser and more open (Fig. 2). Reductions in vegetation density increase visibility, potentially making it easier for predators and prey to detect each other visually from further away. Fire is also likely to affect acoustic and olfactory detection by altering vegetation structure (e.g. leaf fall from trees after a fire may reduce a predator's ability to stalk prey quietly) (Goerlitz, Greif & Siemers, 2008; Fournier *et al*., 2013), and by disturbing or masking scent trails left by prey and predators alike (e.g. Howey & Snyder, 2020). Additionally, fire influences shelter and food availability, with consequences for animal energetics, health, and reproductive success (Fig. 1). The length of time that visibility is heightened, and shelter availability is reduced, after fire depends on the ecosystem, fire severity, fire history, and post‐fire conditions that influence plant regrowth, particularly rainfall (see ‘Moderating factors’ in Fig. 1). In some cases, regenerative plant growth is rapid, and vegetation can be denser than it was pre‐fire within a matter of months post‐fire. Further, the magnitude of structural change varies between vegetation types. For instance, grassland fires often consume all above‐ground plant material, whereas some woody material usually remains after fire in forests and woodlands (Fig. 2). As such, the term ‘recently burnt’ is highly context dependent and should always be interpreted with regard to the specifics of each case study.

We link fire‐induced habitat changes to animal behavioural responses using the predation risk equation of Lima & Dill (1990):

$$P\left(k\right)=1-\text{exp(–α}\mathrm{dT})$$

where predation risk *P*(k) for prey is a function of encounter rate (α), conditional probability of death given an encounter (*d*), and time vulnerable to an encounter (*T*; Fig. 1). As per Hebblewhite, Merrill & McDonald (2005), we use the notation *P*(k), instead of *P*(d), to avoid confusion with the symbol for conditional probability of death (Lima & Dill, 1990). The rate of encounter (when either predator or prey detect the other) can vary with predator movement, density, search behaviour, habitat structure, prey behaviour and other factors. The probability of prey death depends on both the probability of an encounter occurring and the probability that the encounter is followed by the predator successfully capturing and killing the prey. The time spent vulnerable to an encounter depends on factors such as how much time prey spend moving, when they move relative to predators (e.g. diel activity overlap), and where they move relative to predators (e.g. distance from protective cover), amongst others. Related to the predation risk equation are the concepts of habitat‐specific escape ability and habitat‐specific predator lethality (Heithaus *et al*., 2009). Fire can alter the ability of prey to escape from predators, and the ability of predators to catch and kill prey. For a given predator–prey combination, whether the predator, the prey, or neither benefit from fire‐induced habitat changes depends on their specific behaviours and specialisations, which we elaborate on in Sections III, V.

The effects of fire on predator–prey interactions can also be shaped by spatial and temporal scaling (see ‘Moderating factors’ in Fig. 1). Spatial scaling is most relevant at the intersection of fire size and animal body size (Nimmo *et al*., 2019). What is considered a large fire for one species may be a small fire for another. For instance, a 5‐ha fire could encompass numerous home ranges of a 100 g prey species, but might affect only one or a few larger predators (>2 kg) that live in this area. By contrast, a very large fire (e.g. >10000 ha) could impact an entire population of larger predators, as well as several prey populations. Because fire restarts a successional trajectory, or prompts changes to new states, how predators and prey respond is heavily influenced by how much time has elapsed post‐fire (i.e. temporal scaling). In line with optimal foraging theory, a predator or prey species may benefit from fire in the short term (e.g. weeks post‐fire), before changes in food availability or vegetation render the burnt area less beneficial than surrounding unburnt habitat. Consequently, our understanding of these relationships is affected by the time point at which post‐fire data collection occurs. The season in which the fire occurs may also influence how predators and prey respond to fire (e.g. Valentine *et al*., 2007; Braun de Torrez, Ober & McCleery, 2018). For example, Conner, Castleberry & Derrick (2011) suggested that increased time between a fire event and green‐up may result in decreased prey survival due to the existence of a prolonged period of reduced food and shelter availability. Finally, as detailed later, the effect of fire on predator–prey interactions can vary among ecosystem and vegetation types (e.g. Cherry, Warren & Conner, 2017; Cherry *et al*., 2018), due to both inherent structural differences in vegetation and differing inter‐fire intervals, ranging from annual to multi‐century intervals. Hereafter, we draw on the conceptual model in Fig. 1 to answer the four key questions outlined in Section I.

## III HOW AND WHY DO PREDATORS RESPOND TO FIRE?

Predators can respond both numerically and behaviourally to fire (Didham *et al*., 2007; Doherty *et al*., 2015b), which can directly influence encounter rates with prey. A numerical response occurs when predator activity or density changes following fire, whereas a behavioural response may include changes in temporal activity, diet, or hunting behaviour. Importantly, behavioural responses, such as movement and habitat use, can trigger numerical responses, since these behaviours can affect local density or activity in response to fire. If predators are drawn into burnt areas from surrounding unburnt habitat, predator–prey encounter rates may increase by virtue of there simply being more predators in the local area. For instance, it is common for large numbers of raptors to converge at active fires to consume fleeing prey while their shelter is burning (Bonta *et al*., 2017). Predators may also be attracted to recently burnt areas if they are tracking herbivores that move into these areas to feed on flushes of green regrowth post‐fire (Eby *et al*., 2014; Green *et al*., 2015). Texas horned lizards *Phrynosoma cornutum* are thought to target burnt areas due to the abundance of harvester ants *Pogonomyrmex* spp. in these areas, which are their main prey (Fair & Henke, 1997; Hellgren *et al*., 2010). Alternatively, if predator abundance remains the same, but resident predators now concentrate more of their time in burnt areas (e.g. Hradsky *et al*., 2017c), encounter rates *per capita* of resident predators may increase. The *per capita* impact on prey may be exacerbated if prey numbers have been depleted by the fire, and also if predator activity or density is subsidised by an increase in the availability of carrion from animals that were killed in the fire. By the same token, if the density or activity of a predator species decreases in recently burnt areas, encounter rates with prey are likely to decline (e.g. Eby *et al*., 2013).

Hunting strategy can be a key determinant of whether a predator species is more or less abundant or active in burnt areas (Podgaiski *et al*., 2013). Two non‐exclusive hypotheses, the prey catchability hypothesis and the prey abundance hypothesis, have been used to explain variation in predator habitat use and hunting behaviour. The prey abundance hypothesis predicts that predators will choose to hunt where their potential rate of encounter with prey is highest (Litvaitis, Sherburne & Bissonette, 1986; Smith *et al*., 2020). In this case, hunting locations are driven by local prey abundance. By contrast, the prey catchability hypothesis predicts that predators will hunt where their probability of capturing prey given an encounter is highest (Hopcraft, Sinclair & Packer, 2005; Balme, Hunter & Slotow, 2007). By reducing vegetation density, fire can make it easier for predators and prey to detect each other from further away. As such, fire can be beneficial for predators that preferentially forage in open areas, such as pursuit predators, whereas predators that rely on cover for stalking and ambushing prey may be less common in burnt areas. In support of the prey catchability hypothesis, lions *Panthera leo* (typically an ambush predator) in Tanzania avoided burnt areas, even though their prey were abundant there, probably because lions prefer to hunt in areas with high vegetation cover as this favours their short‐range stalk and ambush hunting style (Hopcraft *et al*., 2005; Eby *et al*., 2013). However, a more recent study found that lions increased their use of prey‐rich burnt areas in southern Africa, possibly because the retention of shrubs after fire facilitated hunting (Gigliotti *et al*., 2022). In the Appalachian Mountains, USA, bobcats *Lynx rufus* strongly selected forest edges created by fire and other disturbances, likely because the denser understorey vegetation at the forest edge relative to the interior facilitated their ambush hunting strategy (McNitt *et al*., 2020). By contrast, the abundance of raptors in Oklahoma, USA, increased almost sevenfold during fires as they hunted in the newly opened up habitats (Hovick *et al*., 2017). These opposing responses demonstrate the potential of fire either to decrease or increase encounter rates with prey, depending on predator hunting tactics.

**Fig. 3:**
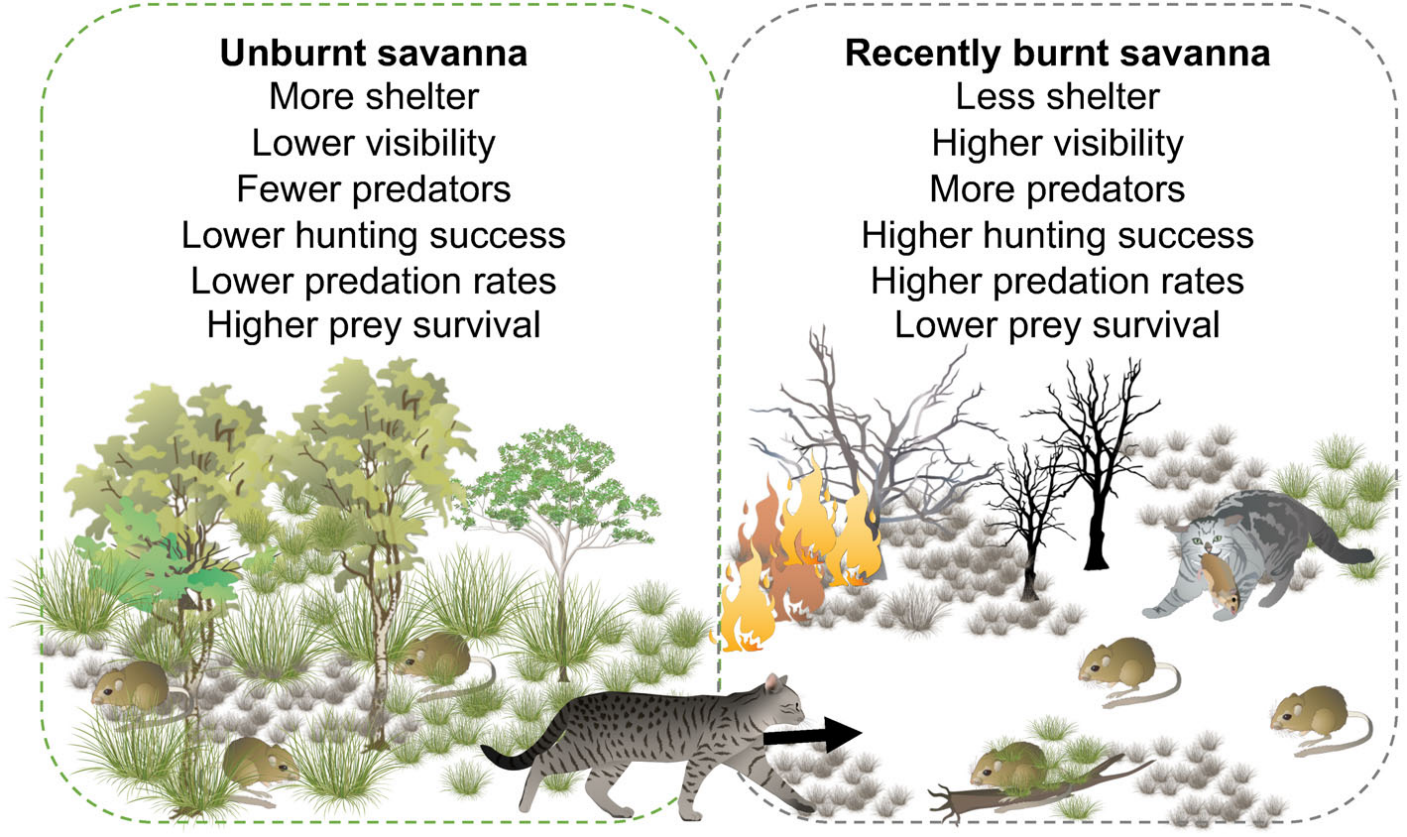
Representation of how fire in tropical northern Australia influences predator and prey behaviour and interactions (McGregor *et al*., 2014, 2015, 2016a,b; Leahy *et al*., 2015). The strength of the effects is highly time dependent because studies found that feral cats used fire scars that were 0–2 (McGregor *et al*., 2016a) or 0–3 months old (McGregor *et al*., 2014) more intensively than older fire scars. Images are courtesy of the Integration and Application Network (ian.umces.edu/media‐library) and the NESP Northern Australia Hub (nespnorthern.edu.au).

In support of both hypotheses, feral cats *Felis catus* in northern Australia were attracted to recently and severely burnt areas with high small mammal abundance (McGregor *et al*., 2014), made long distance forays to reach recent fire scars (McGregor *et al*., 2016b), and had higher hunting success in open compared to complex microhabitats (McGregor *et al*., 2015) (Fig. 3). This represents a case of habitat‐specific predator lethality, whereby cats were more efficient predators in burnt compared to unburnt areas. We are unaware of other studies that have specifically assessed predator hunting success in relation to fire, but studies on habitat structure more generally provide additional evidence about habitat‐specific lethality (Janssen *et al*., 2007). For instance, attack success for Bonelli's eagle *Aquila fasciata* was more than 80 times higher when prey were in open compared to closed habitat (Martínez *et al*., 2014). Hunting success of coyotes *Canis latrans* in dense spruce (mean = 51%) was higher than in sparse spruce (28%), whereas lynx *Lynx canadensis* hunting success was similar between the two (overall mean = 30%) (Murray, Boutin & O'Donoghue, 1995). Capture success of insect prey by bats *Myotis myotis* was almost 100% in sparse and medium‐density vegetation, but only 40% in dense vegetation (Rainho, Augusto & Palmeirim, 2010). When exposed to predation by hedgehogs *Erinaceus europaeus*, daily survival of grasshoppers in low‐complexity grasslands was less than half that of grasshoppers in structurally complex grasslands (Norbury & van Overmeire, 2019). We expect that fire‐induced changes to habitat structure frequently improve predator lethality, thus partly explaining why many predators are attracted to recently burnt areas.

Feral cats in Australia provide a good case study of divergent responses to fire by a single species. McGregor, Cliff & Kanowski (2016a) and McGregor *et al*. (2016b) found that cats in the tropical north travelled long distances (mean = 10.8 km, range = 1–30 km) to visit recent fire scars that were 0–8 months old (Fig. 3). In temperate southern Australia, feral cat occupancy before and after a prescribed burn increased 225% from an average of 0.04 to 0.13 at a burnt site and (20%) from 0.20 to 0.24 at an unburnt control site (Hradsky *et al*., 2017a). In the Simpson Desert, central Australia, cat activity was higher along the ecotone of a burn, compared to areas of either burnt or unburnt habitat away from the edge (Pastro, 2013). However, other work conducted over longer timescales post‐fire or in different ecosystems has found opposite or neutral responses of cats to fire (e.g. Hradsky *et al*., 2017b; Parkins, York & Di Stefano, 2018; Moore *et al*., 2018). In south‐eastern Australia, cat activity was 78% lower ~6–9 months post‐fire compared with pre‐fire activity (Arthur, Catling & Reid, 2012), and on Kangaroo Island, there was a mean 72% decrease in cat detections 5–8 months after a very high severity fire (Hohnen *et al*., 2021). In addition to environmental variation (e.g. fire type, fire severity, vegetation type), temporal scaling is likely to be important for explaining these contrasting responses. Detecting the immediate response of predators to fire requires sampling to occur within hours, days or weeks of the fire (and ideally also pre‐fire), or for animals to be GPS‐tracked to measure their movements and habitat use before, during and after fire. For the two studies that detected post‐fire declines in cat activity 5–9 months post‐fire (Arthur *et al*., 2012; Hohnen *et al*., 2021), it is possible that there were temporary spikes in cat activity within a few months of fire that went undetected. Such variation in how a single species responds to fire makes it difficult to derive general patterns and highlights the importance of developing a mechanistic understanding of predator–prey interactions in response to fire (Fig. 1).

The movement behaviour and territoriality of predators may also affect when and over what spatial scale they exploit burnt areas. For instance, many raptors are highly nomadic and track resources across large areas, meaning they are adapted to responding to fires that occur at unpredictable locations and times (Pavey & Nano, 2013; Bonta *et al*., 2017; Jahn *et al*., 2021). Such movement of predators between burnt and unburnt areas means that fire can alter predator–prey dynamics beyond the fire perimeter. Although untested, forays by predators into burnt areas may temporarily relieve predation pressure on prey in adjacent unburnt areas (McGregor *et al*., 2016b; Bonta *et al*., 2017). In contrast to nomadic raptors, swift fox *Vulpes velox* responses to fire were constrained by territoriality (Thompson, Augustine & Mayers, 2008). Foxes did not shift their home ranges to include more of the burnt area, but individuals whose core range overlapped the burn increased their use of this area for hunting and denning (Thompson *et al*., 2008). A similar pattern was observed for introduced red foxes *V. vulpes* in south‐eastern Australia (Hradsky *et al*., 2017c).

Intra‐guild dynamics can also shape how predators respond to fire (Gigliotti *et al*., 2022). Several studies have reported that subordinate predators used burnt or unburnt areas as a means of predator avoidance. For instance, in semi‐arid Australia red foxes showed no direct response to fire, but were negatively associated with dingoes *Canis dingo* which preferred recently burnt (<11 years post‐fire) areas (Geary *et al*., 2018). In South Africa, lions showed a positive response to prey‐rich burnt areas, but the subordinate predators (spotted hyena *Crocuta crocuta*, cheetah *Acinonyx jubatus*, and leopard *Panthera pardus*) showed a neutral response, suggesting that fire may suppress hunting opportunities for some species due to apex predator avoidance (Gigliotti *et al*., 2022). By contrast, San Joaquin kit foxes *Vulpes macrotis* were more common in burnt areas, possibly because it was easier for them to avoid bobcats and coyotes *Canis latrans* there (Warrick & Cypher, 1998). Similarly, use of burnt areas by gray foxes *Urocyon cinereoargenteus* may have helped them avoid competition with the larger, more dominant coyote which was found more often in unburnt areas (Borchert, 2012). In other studies, predators have responded primarily to the abundance of small mammalian prey post‐fire and have shown little association with each other (e.g. Puig‐Gironès & Pons, 2020).

Shifts in predator diets after fire are common and can reflect changes in both the abundance and availability (e.g. catchability) of different foods. After late dry season fires in northern Australia, the volume of food in the stomachs of frilled‐neck lizards *Chlamydosaurus kingii* almost doubled, which the authors attributed to increased access to prey after grass cover was removed by fire (Griffiths & Christian, 1996). However, reduced competition may also have been important given that the fires killed 29% of radio‐tagged lizards (Griffiths & Christian, 1996). By contrast, fire negatively impacted the diet of bromeliad‐dwelling frogs in Brazil (Rocha *et al*., 2008). At a burnt site, 73% of frogs had empty stomachs, compared to 27% at an unburnt site where the number of prey items per stomach was also higher (Rocha *et al*., 2008). Also in northern Australia, northern quolls *Dasyurus hallucatus* increased their consumption of golden bandicoots *Isoodon auratus* after fire, and golden bandicoots increased their consumption of reptiles (Radford, 2012). The loss of shelter may have provided quolls and bandicoots with improved access to food that was more profitable than the invertebrates that usually dominated their diets. Similarly, in south‐eastern Australia, red foxes increased their consumption of medium‐sized mammals (0.5–7 kg) post‐fire and decreased their consumption of large mammals (10–20 kg; Hradsky *et al*., 2017a). Notwithstanding possible scavenging, this dietary shift suggests that fire may have increased the encounter rate of foxes with medium‐sized mammals, or decreased their encounter rate with larger mammals (Hradsky *et al*., 2017a). In Arizona, USA, gray fox and coyote diets also differed between burnt and unburnt areas, but patterns were not consistent over the 3 years of sampling post‐fire (Cunningham, Kirkendall & Ballard, 2006).

Fire severity and intensity can also have a strong effect on how predators respond to fire. Spotted owls *Strix occidentalis* avoided larger patches of severely burnt forest (Kramer *et al*., 2021) and northern goshawks *Accipiter gentilis* avoided high‐severity burnt areas when roosting and foraging (Blakey *et al*., 2020). By contrast, feral cats actively selected fire scars, but only those created by high‐severity, rather than mild fires, and only fire scars that were recent (<9 months old) (McGregor *et al*., 2014), thus demonstrating how both fire severity and age can be important. In California, USA, carnivore species richness was highest at intermediate levels of fire severity diversity (Furnas, Goldstein & Figura, 2022).

A key determinant of fire severity is the season of burning. For instance, late dry season burns are typically more intense and severe than wet season and early dry season burns (Williams, Gill & Moore, 1998). Mortality of frilled‐neck lizards, which prey on insects, was 0% in early dry season burns, but 29% in late dry season burns (Griffiths & Christian, 1996). Also in northern Australia, ‘edge‐open’ foraging bats responded positively and negatively to low‐ and high‐severity burns, respectively, whereas bats that forage in the open showed a strong positive response to high‐severity burns (Broken‐Brow *et al*., 2020). Similarly, in Florida, USA, bats increased their activity after wet season burns and even more so after dry season burns, likely because of increased availability of insect prey post‐fire (Braun de Torrez *et al*., 2018). Tropical savanna bird abundance also increased after burning, with carnivore abundance highest after wet season burns, and insectivore and granivore abundance highest after dry season burns (Valentine *et al*., 2007).

## IV HOW AND WHY DOES PREY VULNERABILITY CHANGE POST‐FIRE?

**Fig. 4:**
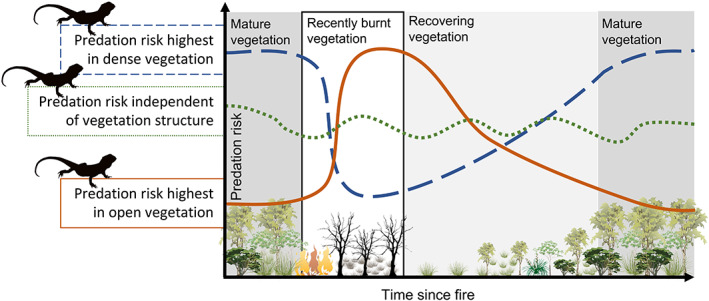
Generalised changes in predation risk in response to fire and vegetation structure. The blue dashed line represents a prey species that experiences lower predation risk when habitat is opened up by fire, the solid orange line represents the opposite response (increased predation risk post‐fire), and the green dotted line represents a prey species for which predation risk is independent of vegetation structure and burning.

Changes in habitat structure after fire can influence both the rate at which prey encounter predators and the conditional probability of prey death (Figs 1, 4). Some prey species benefit from the opening up of habitat after fire because it makes it easier for them to detect and avoid predators, thereby reducing encounter rates (e.g. Jaffe & Isbell, 2009; Cherry *et al*., 2018), whereas other prey rely on habitat structure to reduce predation risk (e.g. Derrick, Conner & Castleberry, 2010; Doherty, Davis & van Etten, 2015a). Species in this latter group are generally disadvantaged by the loss of cover in recently burnt areas and often experience greater predation rates as a consequence (Fig. 4). Leahy *et al*. (2015) found that rodent abundance declined after fire, and predation rates were higher and survival lower in a high‐severity compared to low‐severity burn and an unburnt control (Fig. 3). Wilgers & Horne (2007) similarly found that model snakes placed in burnt habitat had lower daily survival rates than models in unburnt habitat. They concluded that burning tallgrass prairie likely increases predation pressure on large snakes for 1–2 months post‐fire due to lack of cover against aerial predators. Studies on hares *Lepus crawshayi* (Ogen‐Odoi & Dilworth, 1984), mulgaras *Dasycercus blythi* (Körtner, Pavey & Geiser, 2007), and northern quolls (Oakwood, 2000) all found that the majority of known predation events occurred in burnt rather than unburnt areas. In urban bushland remnants, almost all mygalomorph spiders survived a low‐intensity prescribed burn, whereas zero spiders were confirmed alive 12 months after an intense wildfire, possibly due to increased predation (Mason *et al*., 2019). By contrast, flower beetles *Protaetia* spp. were more abundant in burnt compared to unburnt areas, possibly because there were fewer predators in burnt areas (Pausas *et al*., 2018). Taken together, these results point to a key role of predation in shaping prey population dynamics in burnt landscapes, especially because direct mortality rates from fire are often low (Jolly *et al*., 2022).

However, other studies have found no difference in prey survival pre‐ *versus* post‐fire, suggesting that predation rates were not elevated in burnt areas in those instances (e.g. Vernes, 2000; Fig. 4). Similarly, while some studies have found higher rates of nest predation or lower nest survival in burnt compared to unburnt areas (Humple & Holmes, 2006; Churchwell *et al*., 2008; Dziadzio *et al*., 2016; Bahía & Zalba, 2019), others have found mixed or no effects of fire (Hendricks & Reinking, 1994; Gabrey, Wilson & Afton, 2002). Predation rates of artificial nests did not vary with prescribed burning in Georgia, USA, but the dominant predators preying on the nests did, with bird predation dominant in burnt plots and small mammal predation dominant in unburnt plots (Jones *et al*., 2002). Consistent with situations where heightened predator activity post‐fire was relatively short‐lived (e.g. McGregor *et al*., 2016b), increased predation rates post‐fire can also be relatively temporary. Morris & Conner (2016) examined a range of factors affecting predation of more than 1000 artificial nests over 12 years and found that recent burning was the strongest driver. Nests placed in areas burnt less than 2 months earlier were 2.7 times more likely to be depredated than nests burnt 3–23 months earlier (Morris & Conner, 2016).

Alternative prey availability can also mediate predation risk for a prey species (Lannin & Hovel, 2011; Nordberg & Schwarzkopf, 2019). As discussed throughout this review, changes to habitat structure brought on by fire can alter predation risk for some species, but not necessarily others. Predators with flexible diets can therefore take advantage of this, switching focus to the species that are most profitable to target in terms of availability and catchability. For example, in south‐eastern Australia, red fox diet changed to include a lower proportion of large‐sized mammals (e.g. wallabies) and a higher proportion of medium‐sized mammals (e.g. bandicoots, echidnas) after a prescribed burn (Hradsky *et al*., 2017a). Additionally, after large, severe fires which cause high prey mortality, the most available food source may be carrion and so predators may preferentially scavenge post‐fire rather than pursue live prey (Newsome & Spencer, 2021). Therefore, some prey species may be less susceptible to predation post‐fire if there are more abundant or more available food sources that predators can exploit.

Probability of prey death given a predator encounter may be lower in burnt areas if prey are able to detect predators from further away. For instance, after fire, vervet monkeys *Cercopithecus aethiops* ranged further from trees that provide refuge from mammalian predators (Jaffe & Isbell, 2009). Visibility was 10 times higher in burnt areas, and monkeys travelled faster and spent less time scanning and more time feeding (Jaffe & Isbell, 2009). Another study on a different species of vervet monkey *C. pygerythrus* found that predator‐related behaviours (e.g. vigilance, fleeing) rarely occurred in burnt areas, suggesting that monkeys feel safer there, perhaps due to increased visibility (Herzog *et al*., 2020). On the other hand, arboreal species that are less well adapted to moving along the ground may be particularly vulnerable after fires (Laurance, 2003). Fire can reduce canopy resources and connectivity, which may force arboreal species to come to ground more often, as seen elsewhere when resource availability was reduced (e.g. Souza‐Alves *et al*., 2021). Short‐term survival of ringtail possums *Pseudocheirus peregrinus* reintroduced to a park 4 years after fire was much lower than that for animals released 3–4 years before the fire (Russell, Smith & Augee, 2003). Decreased canopy and understorey continuity after fire caused possums to nest more frequently in tree hollows, rather than in dreys, which was thought to increase their exposure to reptilian predators (Russell *et al*., 2003).

Reductions in animal health and body condition post‐fire may also make prey more vulnerable to predation (Wirsing, Steury & Murray, 2002). Animals that survive fire often must cope with exposure to the elements and reduced food availability, with possible negative impacts on body condition, fecundity and survival (Morris *et al*., 2011b). In Australia, pygmy bluetongue lizards *Tiliqua adelaidensis* at burnt sites reduced their activity and had lower body condition relative to unburnt sites (Fenner & Bull, 2007). This may have been due to a greater exposure to predators from reduced grass cover, stress following the fire event, or lower food availability as the lizards are ambush predators that rely on prey crossing their burrow entrance (Fenner & Bull, 2007). Bush rats *Rattus fuscipes* in Australia (Fordyce *et al*., 2016) and bats in Italy (Ancillotto *et al*., 2021) also experienced decreased body condition post‐fire. Such decreases in body condition can increase predation risk through two main mechanisms. Firstly, energetically stressed animals may be less effective at escaping predation events due to reduced locomotory performance (Alzaga *et al*., 2008; Zamora‐Camacho *et al*., 2015; Rew‐Duffy *et al*., 2020). Secondly, state‐dependent risk taking may make energetically stressed animals more likely to forage in risky areas because the potential rewards are greater for them compared to animals in better body condition (Godfrey & Bryant, 2000; Heithaus *et al*., 2007; Berger‐Tal *et al*., 2010). Such behaviour could increase a prey animal's encounter rate with predators and the time they are vulnerable to an encounter (Fig. 1). By contrast, some studies found no change or an increase in body condition but also noted that their study species did not face difficulties locating food following the fires (Lovich *et al*., 2011; Lewis *et al*., 2012; Lecq *et al*., 2014; Cole & Hataway, 2016; Smith, 2018) and had microhabitats available for refuge (Lovich *et al*., 2011; Lecq *et al*., 2014). As discussed in Section V, reduced body condition may also be directly related to increased predation risk in the post‐fire environment when prey decrease their foraging activity to reduce exposure.

## V WHAT MECHANISMS DO PREY USE TO REDUCE PREDATION RISK POST‐FIRE?

In response to heightened risk of predation in recently burnt habitat, many prey species alter their habitat use for foraging, resting, and predator avoidance. For example, in the forests of south‐eastern Australia, bush rats avoided burnt vegetation in favour of unburnt patches, increased their use of structurally complex habitat, and made more convoluted movements when foraging (Fordyce *et al*., 2016; Lees *et al*., 2022). The authors suggested that these behavioural changes were, at least in part, driven by heightened levels of perceived predation risk in burnt forest. Similarly, in Alaska, USA, caribou *Rangifer tarandus* avoided the interior of burns, used the interior and exterior edges of burns (within 500 m) in proportion to their availability, and selected unburnt areas more than 500 m from burns (Joly *et al*., 2003). Although not related to fire, ungulates in an African savanna visited open sites where shrubs were experimentally removed 2.4 times more frequently than shrubby control sites (Epperly *et al*., 2021). The ungulates were 47% more likely to flee from carnivore vocalisations in shrubby control sites compared to open sites, indicating they felt safer in open habitat (Epperly *et al*., 2021).

**Fig. 5:**
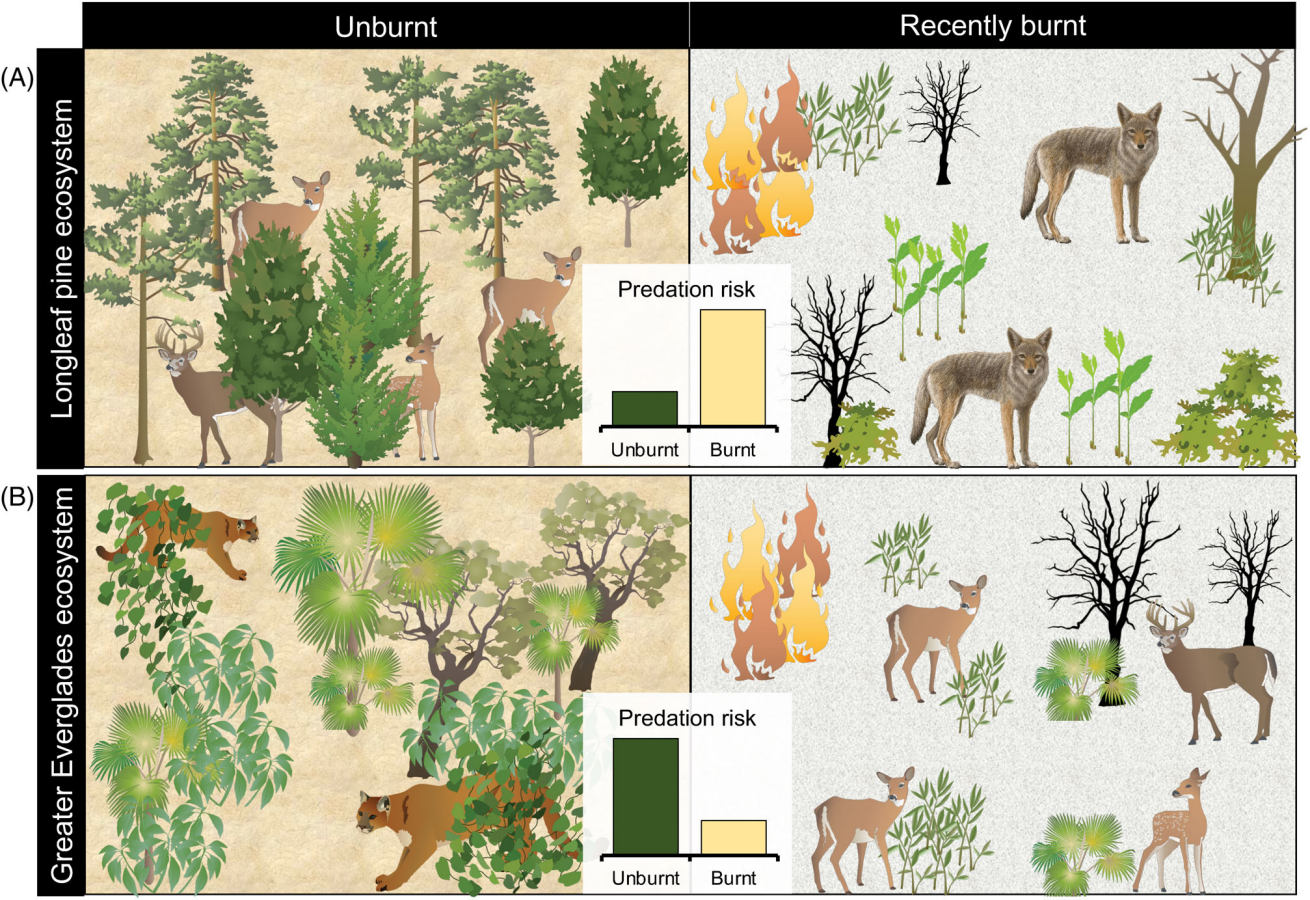
Contrasting responses of white‐tailed deer *Odocoileus virginianus* to fire‐induced changes in predation risk in two ecosystems. (A) In longleaf pine woodlands in Georgia, USA, deer avoided recently burnt areas, despite forage availability being higher there, most likely to reduce risk of predation by coyotes *Canis latrans* (Cherry *et al*., 2017). (B) In the Greater Everglades, Florida, USA, deer increased their use of a burnt area to capitalise on increased food availability and lower risk of predation by Florida panthers *Puma concolor coryi*, which use cover for ambushing prey (Cherry *et al*., 2018; Abernathy *et al*., 2022). Images are courtesy of the Integration and Application Network (ian.umces.edu/media‐library).

In Georgia, USA, white‐tailed deer *Odocoileus virginianus* also avoided recently burnt areas, despite an abundance of food in those areas (Fig. 5A; Cherry *et al*., 2017). Using a foraging experiment that standardised food availability between burnt and unburnt areas, the authors showed that deer foraging activity increased with time since fire. They concluded that avoidance of recently burnt areas with reduced cover is due to an increased risk of predation by coyotes, which are cursorial predators (Cherry *et al*., 2017). By contrast, Cherry *et al*. (2018) found that white‐tailed deer in Florida, USA, increased their use of an area following a wildfire by an average of 10–19%, relative to pre‐fire and depending on the month (Fig. 5B). In a longer‐term study occurring at the same Florida site, Abernathy *et al*. (2022) observed female white‐tailed deer that survived the fawning season preferred areas that were both recently and frequently burnt. In both studies, the authors concluded that fire in this system not only improves food availability but may also reduce predation risk from the Florida panther *Puma concolor coryi*, which is an ambush predator that relies on cover for stalking prey. However, Dees, Clark & Manen (2001) radio‐tracked panthers in the same area and found that panthers actively selected for management burns less than 1 year old, which the authors suggested was due to increased prey abundance. The different conclusions reached by these two studies may be due to differences in burn characteristics and vegetation structure, particularly given that one study focused on prescribed burns and the other on wildfire. Additionally, it is worth noting that the first study tracked deer, whereas the second tracked panthers. Taken together, these findings suggest that panthers are responding to prey abundance, while deer likely experience reduced *per capita* predation risk in burnt areas due to decreased catchability and increased local abundance (Dees *et al*., 2001; Cherry *et al*., 2018; Abernathy *et al*., 2022).

For prey that rely on habitat structure to reduce predation risk, the amount of time they are vulnerable during a predator encounter can depend on shelter availability (Fig. 1). When these prey forage further away from shelter, it takes them longer to reach refuge when pursued by a predator. Prey can reduce their exposure time through risk‐sensitive foraging, such as by being more vigilant or foraging closer to shelter (Abramsky *et al*., 1996; Doherty *et al*., 2015a). For instance, GPS tracking of wild turkeys *Meleagris gallopavo* in Louisiana, USA, found that the probability of use of recently burnt areas decreased as distance to escape cover increased (Yeldell *et al*., 2017). However, this effect decreased with increasing time since fire, possibly due to vegetation recovery that reduced predation risk in burnt areas. Another study similarly found that turkey habitat use decreased with increasing distance from unburnt vegetation and that turkeys were more likely to be walking rather than foraging in the interior of burnt areas (Cohen *et al*., 2019). Walking behaviour was characterised by higher speeds and straighter movements than foraging behaviour (Cohen *et al*., 2019). It is unclear whether this behavioural shift in burnt areas is related to predation risk or food availability, but faster and more directed movements in burnt areas could reduce the time birds are vulnerable to predators when an encounter occurs.

Prey species may also vary their foraging strategies temporally, as well as spatially, to decrease the risk of predation. For example, in South Africa, Burkepile *et al*. (2013) examined the diurnal and nocturnal foraging patterns of several ungulate species in unburnt areas and areas burnt either annually or triennially. Zebras *Equus quagga* foraged in areas burnt annually or triennially during the day, but avoided triennially burnt areas at night, as they contained more dense woody vegetation favouring ambush predators (e.g. lions), which are more active at night (Funston, Mills & Biggs, 2001). Similarly, impala *Aepyceros melampus* foraged in triennially burnt and unburnt areas during the day, but used less risky areas at night (i.e. annually burnt areas) when the risk of lion predation was greater (Burkepile *et al*., 2013). In Australia, two small mammal species decreased their daytime activity and increased their use of torpor in recently burnt compared to unburnt areas (Stawski *et al*., 2015; Matthews *et al*., 2017). It was thought that this strategy was used to conserve energy and avoid foraging in open habitat where predation risk could be higher. By one‐year post‐fire, when there had been some vegetation recovery, torpor use and activity in the burnt area had returned to levels recorded pre‐fire and in unburnt control areas (Stawski *et al*., 2017). Importantly, torpor saves fat stores and studies have found that individuals will employ torpor even when in good body condition, hypothesising that this reduces the risk of predation by allowing animals to restrict foraging to when the benefits are greatest (Stawski & Geiser, 2010; Turbill, McAllan & Prior, 2019).

Cryptic colouration and background matching can also help prey reduce their risk of predation in burnt areas (Endler, 1978; Forsman *et al*., 2011; Pausas & Parr, 2018). This is a phenomenon whereby burnt environments act as a selective force for dark colouration by reducing an animal's chance of being detected by a predator in fire‐blackened areas (Guthrie, 1967; Karlsson *et al*., 2008; Forsman *et al*., 2011). For example, in Sweden, the frequency of the melanistic morph of the pygmy grasshopper *Tetrix subulata* was higher in populations occupying recently burnt compared to unburnt areas (46% *cf*. 9%) (Forsman *et al*., 2011). The proportion of melanistic individuals declined to 33% in the proceeding 4 years after fire because the grasshoppers have short lifespans (~12 months), and dark body colour no longer provided the same survival benefit once vegetation recovered. To test whether melanistic colouration reduces the risk of predation by visual predators post‐fire, Karpestam, Merilaita & Forsman (2012) presented images of black grasshoppers against different backgrounds (0–100% of background burnt) to human subjects acting as predator analogues. The chance of not being detected and the average survival time (i.e. time taken to detect) was approximately three to four times higher in the completely burnt compared to the unburnt images. Similarly, populations of the eastern fox squirrel *Sciurus niger* occurring in the fire‐prone region of south‐eastern USA exhibit darker colouration (Kiltie, 1992b; Potash *et al*., 2020), which provides superior camouflage against a variety of backgrounds in fire‐prone ecosystems (Kiltie, 1992a,b). In chaparral of southern California, USA, the darkly coloured western fence lizard *Sceloporus occidentalis* selectively perched on the blackened stalks of burnt shrubs for several years after fire (Lillywhite, Friedman & Ford, 1977). Notably, research on the evolutionary aspects of background matching in prey species in fire‐prone environments is still in its infancy, with many of the phenotypic responses yet to undergo rigorous testing (Pausas & Parr, 2018).

## VI WHAT ARE THE OUTCOMES OF PREDATOR–FIRE INTERACTIONS FOR PREY POPULATIONS?

Experiments where both predator densities and fire are manipulated can provide the strongest evidence regarding the impacts of predator–fire interactions on prey population dynamics. A series of studies from Georgia, USA, monitored the response of small mammal populations to prescribed fire both inside and outside of predator exclosures (Conner *et al*., 2011; Morris *et al*., 2011a,b; Karmacharya *et al*., 2013). The fences excluded most mammalian carnivores, but reptiles and birds were still present. For the hispid cotton rat *Sigmodon hispidus*, mesopredator exclusion improved survival before prescribed fire, but post‐fire survival rates were similar between predator treatments (Conner *et al*., 2011). This likely occurred due to compensatory predation by raptors post‐fire when vegetation cover was lower. Post‐fire predation affected half of the animals being monitored at the time (Conner *et al*., 2011). A related study spanning a longer time period found that prescribed burns reduced hispid cotton rat survival and abundance, presumably by reducing cover and increasing predation risk by non‐mammalian predators (Morris *et al*., 2011a). Predator exclusion had no effect. Cotton mouse *Peromyscus gossypinus* survival increased in predator exclosures following burns, but was stable outside exclosures (Morris *et al*., 2011b). Oldfield mouse *P. polionotus* survival and abundance was higher in response to predator exclusion (Morris *et al*., 2011b). After fire, survival increased slightly inside and decreased slightly outside exclosures (Morris *et al*., 2011b). Finally, for the southern flying squirrel *Glaucomys volans*, prescribed fire increased survival, but there was no apparent effect of food supplementation or predator exclusion (Karmacharya *et al*., 2013). In that case it was unclear if there was compensatory predation by raptors and snakes inside exclosures. The positive effect of fire may have been due to reduced understorey vegetation, making movement and predator detection and avoidance easier (Karmacharya *et al*., 2013).

Population modelling for the northern bettong *Bettongia tropica* in Australia showed that predation by feral cats had a strong impact on population viability, which became stronger in the presence of fire (Whitehead *et al*., 2018). Low levels of cat predation reduced population size by 8–18.5%, but the probability of extinction was 0%. Adding fire caused declines in abundance of 30–62% relative to the baseline, yet the probability of extinction was still very low (<1%). At moderate and high levels of cat predation, the interactive effect of fire was much weaker, and probability of extinction was 100% for all scenarios, irrespective of fire. Adding fire hastened time to extinction by 3–4 years for the moderate predation scenario, but had almost no impact with high predation (Whitehead *et al*., 2018). Similarly, Lunney *et al*. (2007) showed that reducing or eliminating predation by dogs had a stronger positive effect on koala *Phascolarctos cinereus* population growth rate (*r*) and survival than did reducing or eliminating fire. Eliminating both fire and dog predation was the only scenario that resulted in a positive population growth rate and the effect appears additive, rather than synergistic, because the increase in population growth rate (0.29) relative to the baseline was similar to the increase when summing the two independent scenarios (0.28; Lunney *et al*., 2007).

Long‐term population studies of the splendid fairy‐wren *Malurus splendens* also revealed how fire and predation influence population viability (Rowley & Brooker, 1987; Rowley, Brooker & Russell, 1991; Russell & Rowley, 1993; Brooker & Brooker, 1994). After a major fire affected the study population in 1985, the rate of nest predation increased to 37.3% in 1985–88 compared to 18% in 1973–77 and 20% in 1978–84 (Rowley *et al*., 1991). No birds were known to die in the fire and adult survival in the following years was not affected (Rowley & Brooker, 1987; Russell & Rowley, 1993). Population modelling showed that increasing rates of nest predation related to fire led to lower recruitment rates and, when combined with the effects of brood parasitism and rainfall, could cause the near extinction of the population (Brooker & Brooker, 1994).

## VII CONSERVATION AND MANAGEMENT IMPLICATIONS

Although fire has affected predator–prey interactions for millennia (Hoare, 2019), human modification of Earth's ecosystems now means that the intersection of fire with predator–prey dynamics can have unexpected and undesirable outcomes. Fire regimes globally have shifted from historical baselines due to the displacement of Indigenous Peoples (Bird *et al*., 2020; Mariani *et al*., 2022), climate change (Halofsky, Peterson & Harvey, 2020), increased rates of prescribed burning (Fernandes, 2018; Cirulis *et al*., 2020) or fire suppression (Schmidt & Eloy, 2020), land‐use change (Chergui *et al*., 2018), and habitat change such as the introduction of invasive grasses that initiate a grass‐fire cycle (D'Antonio & Vitousek, 1992; Setterfield *et al*., 2010). Changes in the frequency, size, intensity and season of fire in many ecosystems are exposing faunal communities to novel fire regimes, with consequences for predator and prey behaviour and interactions.

Increases in fire activity, such as that caused by climate change, invasive grasses, land‐use change and prescribed burning, may create more open habitat in the short term, with fewer shelter sites and increased visibility. Repeated fires may lead to continued simplification of habitat structure (Russell‐Smith, Edwards & Price, 2012; Costa *et al*., 2020), or paradoxically create more structurally complex habitat by promoting dense under‐ and mid‐storey regrowth (Foster *et al*., 2017; Borden, Duguid & Ashton, 2021). Intensification of fire regimes is likely to benefit pursuit predators if there is less shelter for prey to escape to. High‐severity fire may temporarily make it easier for ‘open habitat’ prey species to detect and escape from predators (Jaffe & Isbell, 2009), but any survival advantage in terms of predator detection may be offset by concomitant impacts of fire on resource availability, animal health and recruitment (Griffiths & Brook, 2015; Stillman *et al*., 2021). Correspondingly, we expect ambush predators to be disadvantaged by increases in fire frequency, severity or other fire characteristics that reduce the amount of vegetation cover that can be used for concealment while hunting (e.g. Cherry *et al*., 2018).

Tropical northern Australia provides a good example of how changes in fire regimes may be disrupting predator–prey dynamics. Native mammal communities in the high‐rainfall belt of this region coexisted with feral cats for around 100 years before many populations across the region experienced precipitous declines beginning in the late 1980s, leading to local extinctions (Woinarski *et al*., 2010). Increases in fire size, frequency and severity, along with increased grazing pressure and lethal control of dingoes, are thought to have exacerbated the impacts of cat predation on native mammals (Woinarski *et al*., 2011; Leahy *et al*., 2015; Radford *et al*., 2015; Legge *et al*., 2019).

Globally, fire is spreading into ecosystems where it was not common previously, such as some rainforests and tundra vegetation (McCarty, Smith & Turetsky, 2020; Armenteras *et al*., 2021; Godfree *et al*., 2021), and prey species there are likely to be naïve to the interaction between predators and fire (Nimmo *et al*., 2021). Although untested, post‐fire predation on naïve prey may inflict unsustainable levels of mortality, thus hampering population recovery post‐fire. This may be especially important for smaller or less‐mobile taxa, such as small non‐volant mammals, reptiles and some invertebrates. For instance, there is growing evidence that recovery of many small mammal populations after fire is driven by *in situ* survivors, rather than immigrants from outside the fire ground (Banks *et al*., 2017; Shaw *et al*., 2021; Hale *et al*., 2022). Elevated post‐fire predation on these *in situ* survivors could disrupt historical patterns of recovery, such that immigration of individuals from external unburnt habitat becomes more important for population recovery (e.g. Puig‐Gironès, Clavero & Pons, 2018; Shaw *et al*., 2021). The spread of fire into ecosystems where it was previously rare or absent may also disrupt the visual, auditory and olfactory cues that predators rely on for locating and capturing prey, potentially reducing their hunting efficiency (Howey & Snyder, 2020).

Some ecosystems are experiencing decreases in fire activity, either through active fire suppression, land‐use change or climate change (Rogers *et al*., 2020). When fire is suppressed or excluded for long periods of time, it may promote the retention of older and more structurally complex habitat, thus decreasing visibility and increasing the availability of shelter. Very long‐term exclusion of fire could also lead to senescence of mature vegetation and decreases in habitat structural complexity (Gosper *et al*., 2011). In the USA, the combination of fire suppression and drought likely facilitated the encroachment of the invasive eastern red cedar *Juniperus virginiana* into oak *Quercus* spp. forests, leading to increases in forest stand density and structural complexity (DeSantis *et al*., 2010; DeSantis, Hallgren & Stahle, 2011). In parts of Brazil, fire suppression is transforming tropical savannas into forests (Stevens *et al*., 2017). In these cases, the increase in woody vegetation and structural complexity alters resource availability (such as food and shelter) and likely influences animal space use and assemblages, with consequences for predator–prey dynamics. Decreased fire activity may disadvantage pursuit predators that hunt most efficiently in open areas, and benefit ambush predators that use cover for hunting and prey species that rely on complex habitat to reduce predation risk.

While fire is necessary for the maintenance of many natural predator–prey systems, it can facilitate damaging ecological impacts where invasive predators are present. The impacts of introduced predators on vertebrate fauna have generally been greatest on islands where native prey are naïve to the threat of exotic predators (Salo *et al*., 2007; Doherty *et al*., 2016). Fire can exacerbate the impacts of introduced predators, leading to population declines of native prey (Doherty *et al*., 2015b; Legge *et al*., 2019; Hradsky, 2020). Evidence for this phenomenon is almost exclusively limited to red foxes and feral cats in Australia (e.g. McGregor *et al*., 2016b; Hradsky *et al*., 2017a). However, there is strong potential for this dynamic to play out in other locations where fire occurs and naive prey are vulnerable to invasive predators, such as Madagascar (Farris *et al*., 2017), New Caledonia (Palmas *et al*., 2017), and Hawaii (Hess, 2016). Management approaches for reducing invasive predator impacts in relation to fire include conducting low‐severity burns that retain natural refuges (Leahy *et al*., 2015; Shaw *et al*., 2021), providing artificial refuges post‐fire (Bleicher & Dickman, 2020; Watchorn *et al*., 2022), and conducting lethal control of predators, either through long‐term landscape‐scale suppression, or through targeted control at high‐priority sites pre‐ or post‐fire (Comer *et al*., 2020; Hradsky, 2020).

## VIII OUTSTANDING QUESTIONS AND FUTURE DIRECTIONS

Here, we outline key knowledge gaps and outstanding research questions which, if addressed, would greatly enhance our understanding of how fire shapes predator–prey interactions. A key challenge for the field has been in reconciling conflicting results relating to a single species or location. The intersection of different species, ecosystems and fires makes it difficult to derive general patterns, but our conceptual model (Fig. 1) helps identify the mechanisms that lead to different outcomes. For instance, explicitly considering the timescale of sampling relative to when a fire occurred sheds light on the varying results from studies on feral cats (e.g. Arthur *et al*., 2012; McGregor *et al*., 2014, 2016b; Hradsky *et al*., 2017a,b; Hohnen *et al*., 2021). Future studies could focus on capturing data in the hours, days and weeks pre‐ and post‐fire to help understand the immediate impacts of fire on predator–prey behaviour. This is likely to be most feasible for planned burns where the general time and location of the fire is known in advance, thus facilitating strategic and timely data collection. Responding quickly to unplanned fires is more difficult because it can be difficult to mobilise field resources at short notice and access to severely burnt areas may be restricted due to safety concerns. However, long‐term studies in fire‐prone areas can allow researchers to study the effects of unplanned fires (e.g. Russell & Rowley, 1993; Brooker & Brooker, 1994), thus additional support for long‐term research and monitoring projects should be a priority. Another fruitful approach would be to conduct meta‐analyses or other quantitative syntheses focused on a specific taxonomic group, region or phenomenon (e.g. Geary *et al*., 2020; Jolly *et al*., 2022). Synthesis of future studies would also be aided by standardising how fires, the predator and prey guilds, and environmental context are described, so that heterogeneity between study contexts and designs can be accounted for (Geary *et al*., 2020).

Several studies discussed herein have revealed that physiological traits related to energy management, which directly impact predator–prey interactions, can change shortly after a fire and remain so for several months, often not returning to post‐fire levels until some vegetation has returned (Stawski *et al*., 2017). However, these physiological responses to fire vary among species and can be related to factors such as preferred habitat, foraging behaviour, food source and whether they are the predator (e.g. Stawski *et al*., 2015; Doty *et al*., 2016). Studies on physiological traits at the individual level are scarce because such data are difficult to collect, especially regarding unpredictable wildfire. Nonetheless, we urge that additional research focuses on physiological responses of predators and prey to fire. Such research can provide valuable information for designing appropriate fire‐management strategies to promote sustainable predator–prey interactions (Stawski & Doty, 2019).

A key assumption of our conceptual framework and much of the literature is that altered visibility post‐fire is a major driver of prey and predator behaviour. Directly quantifying changes in visibility from the perspective of animals is very difficult and as such there are almost no direct tests of this idea (but see Jaffe & Isbell, 2009). Developing novel means of measuring changes in visibility post‐fire could help unravel the mechanisms more clearly, including separating out the relative importance of visual obstructions compared to provision of shelter or obstacles which prey can use to escape predation when being pursued (e.g. Wheatley *et al*., 2020). Terrestrial LiDAR (Light Detection and Ranging) scanning is one approach that could be used to create three‐dimensional images of vegetation structure pre‐ and post‐fire and assess changes in visibility at different scales (Olsoy *et al*., 2015; Lecigne, Eitel & Rachlow, 2020). Additionally, we have focused on visual encounters between predators and prey themselves, but fire could also make it easier for predators to detect the signs of prey, such as their tracks or shelter sites, thus aiding hunting efficiency. On the other hand, fire may make prey movements less predictable if the removal of vegetation cover reduces the energetic benefit of using well‐worn game trails for movement. Computer‐based research techniques such as individual‐ or agent‐based models (e.g. Wheatley *et al*., 2020), or ‘games’ involving human predators (e.g. Karpestam *et al*., 2012) could help answer these questions.

Related to this is the dearth of information available about how fire affects non‐visual cues. Many predators and prey use olfactory cues to detect and find or avoid one another (Bytheway, Carthey & Banks, 2013), but we do not know how fire affects scent‐mediated behaviours. Conceivably, fire could impair hunting behaviour of ambush predators, such as many snakes, in the short term by destroying the scent trails laid down by prey (Howey & Snyder, 2020). Additionally, there is little information available regarding how fire affects acoustic information. Conceivably, the removal of leaf litter and ground‐level vegetation could allow predators and prey to move around more quietly, thus reducing their detectability (Goerlitz *et al*., 2008; Fournier *et al*., 2013). On the other hand, canopy scorch in forests and woodlands can lead to mass leaf drop in the days and weeks after fire, which may make predator and prey movements noisier (Goerlitz *et al*., 2008; MacLeod *et al*., 2019). Attaching miniature acoustic tags to predators and prey would be a novel means of documenting how fire affects the soundscape of predator and prey movements and interactions (Greif & Yovel, 2019; Studd *et al*., 2021).

The available literature primarily focuses on bipartite interactions (but see Geary *et al*., 2018), but we know that predators and prey live and operate within complex trophic networks. Recent work has examined how carnivore communities respond to fire (Jorge *et al*., 2020; Furnas *et al*., 2022; Gigliotti *et al*., 2022), but there has been little focus on interactions between multiple predator and prey species. A key area for future development is understanding how fire alters networks of interactions between predators and consumers (e.g. Ponisio, 2020). Such information will aid in predicting how predator–prey interactions across a community may shift in response to changing fire regimes (Santos & Cheylan, 2013). Approaches such as ecosystem models and multi‐species occupancy models may be able to assist with this, so that both the direct and indirect effects of fire on predator–prey interactions can be understood.

Predator–prey research to date has mostly focused on binary comparisons of burnt and unburnt habitat, with little focus on specific fire regime characteristics, such as fire size, frequency, severity, and season (Geary *et al*., 2020). In general, we expect that complex ecological interactions such as predator–prey dynamics are influenced by both historical and current aspects of fire regimes, as well as how these interact with antecedent conditions such as climate, co‐occurring disturbances, and other environmental characteristics. For example, historically, prey species could likely withstand increased predation pressure post‐fire because there was ample time between fires for populations to recover. However, in systems where the fire return interval (or other aspects of the regime) is deviating from the long‐term average, instances of amplified predation pressure may be occurring too frequently, leading to population declines. This mechanism, and many others, can only be understood if studies of individual fires are placed in the context of the overall fire regime. As such, a particularly valuable approach for future research will be to couple studies of how individual animals respond to fire events in the short term (e.g. behaviour and mortality) with longer term studies focused on population‐level responses to fire regimes.

As with most fields of ecology, most predator–prey research has focused on mammalian taxa, particularly rodents, canids and felids, and there has been less focus on birds, reptiles, amphibians and invertebrates (Geary *et al*., 2020). These widespread taxonomic biases limit our ability to make predictions about the effect of fire on predator–prey interactions more generally. Similarly, the literature in this space is biased towards North America, Australia, and to a lesser degree Europe (Geary *et al*., 2020). There are relatively few studies from Africa, Asia and South America. Given that fire regimes are intensifying in many regions (e.g. Armenteras *et al*., 2020), we recommend that future research focuses on the impacts of fire on predators and prey in understudied regions to generate new knowledge that can inform ecosystem management and species conservation, including on islands where fire may exacerbate invasive predator impacts.

## IX CONCLUSIONS

1. Fire influences predator–prey interactions through changes in vegetation structure, resource availability, visibility, and acoustic, olfactory and visual cues.
2. How predators respond to fire can be influenced by their hunting behaviour, territoriality, intra‐guild dynamics, vegetation type, fire characteristics, and how much time has elapsed post‐fire.
3. Prey species that depend on dense habitat structure to reduce predation risk often experience increased predation rates following fire, whereas some other prey species benefit from the opening up of vegetation because it makes it easier to detect predators and escape predation events.
4. Prey reduce predation risk by altering their movements, habitat use, foraging, camouflage and other behaviours, which in turn reduces encounter rates with predators, the amount of time they are vulnerable during an encounter, and the probability of death given an encounter.
5. Human‐induced changes to fire regimes globally are likely to alter predator–prey dynamics through changes in behaviour, resource availability and community composition. Given the strong influence that predator–prey interactions have on population dynamics, we urge for increased management and research focus on this issue, particularly in ecosystems where fire was historically not a dominant force.
